# Clinical characteristics of 14 COVID-19 deaths in Tianmen, China: a single-center retrospective study

**DOI:** 10.1186/s12879-021-05770-z

**Published:** 2021-01-20

**Authors:** Jijia Hu, Yingang Zhang, Wei Wang, Zhihe Tao, Juan Tian, Ning Shao, Nian Liu, Hui Wei, Hao Huang

**Affiliations:** 1 Division of Nephrology, Renmin Hospital of Wuhan University, Hubei, China; 2 Division of Medical Services, The First People’s Hospital of Tianmen, Tianmen, Hubei China; 3 Division of Health Examination Center, The First People’s Hospital of Tianmen, Tianmen, Hubei China; 4 Director’s Office, The First People’s Hospital of Tianmen, Tianmen, Hubei China; 5 Division of Rheumatology, The First People’s Hospital of Tianmen, Tianmen, Hubei China; 6 Division of Nephrology, The First People’s Hospital of Tianmen, Tianmen, Hubei China; 7 Division of Cardiology, The First People’s Hospital of Tianmen, Tianmen, Hubei China

**Keywords:** COVID-19, SARS-CoV-2, Deaths, Characteristics

## Abstract

**Background:**

The treatment of critically ill patients with COVID-19 who were hospitalized in Wuhan has been reported. However, the clinical characteristics of patients who died of COVID-19 in regions with relatively scarce healthcare resources remain unknown.

**Methods:**

In this retrospective study, a total of 14 patients who were admitted from January 18 to February 11, 2020 and died of COVID-19 were evaluated. The epidemiological, symptomatic, laboratory, radiological and treatment records were reviewed and analyzed.

**Results:**

The mean age of the 14 patients was 56.7 (SD 15.3) years, and 8 (57.1%) were older than 50 years. Eight (57.1%) were men, and 11 (78.6%) had one or more high risk factors. The most common chronic diseases among these patients were cardiovascular disease (7, 50.0%), hypertension (6, 42.9%), and chronic kidney disease (5, 35.7%). General symptoms included cough (12, 85.7%), fever (11, 78.6%), and dyspnea (10, 71.4%). The median duration from the onset of symptoms to death was 11 (IQR 6.5–19.5) days, and the median duration from admission to death was 4.5 (1.0–11.8) days. Patients who died within 4.5 days had more severe pulmonary lesions, significantly reduced lymphocytes and elevated C-reactive protein (CRP). Most patients had organ dysfunction, including 13 (92.9%) with acute respiratory distress syndrome (ARDS), 4 (28.6%) with cardiac injury, 3 (21.4%) with acute kidney injury, and 3 (21.4%) with liver dysfunction.

**Conclusions:**

Elderly SARS-CoV-2-infected patients with comorbidities, especially those with ARDS and severe chest CT findings on admission, are at increased risk of death and deserve special attention and quality medical treatment.

**Supplementary Information:**

The online version contains supplementary material available at 10.1186/s12879-021-05770-z.

## Background

Coronavirus disease 2019 (COVID-19) caused by severe acute respiratory coronavirus 2 (SARS-CoV-2) is a newly recognized infectious illness in Wuhan, Hubei, that has spread rapidly to numerous countries [1]. As of March 24, 2020, the total number of affected cases has risen to 372,757 globally, with 16,231 deaths [2]. The clinical spectrum of COVID-19 ranges from mild to critical, and the worse prognoses mostly occur in elderly patients with underlying diseases [3]. Previous studies have described the clinical characteristics, diagnosis and treatment processes, and outcomes of patients with COVID-19 who were hospitalized in major cities [3, 4]. However, the characterization of critically ill patients and nonsurvivors in areas with relatively scarce healthcare resources remains unknown.

As the only designated center for the treatment of COVID-19 patients with critical illness in Tianmen City, as of March 6, 2020, a total of 415 SARS-CoV-2-positive patients had been admitted to the First People’s Hospital of Tianmen, and all 14 deaths from COVID-19 in this city occurred at the hospital. We reported that the case-fatality rate (CFR) of COVID-19 in Tianmen was much higher than that in core cities in the early stage of the pandemic [5]. However, specific reports characterizing COVID-19-associated deaths in Tianmen are lacking. To facilitate the scientific diagnosis and treatment of COVID-19 patients with critical illness globally, we retrospectively investigated and analyzed the medical records of 14 patients who died of COVID-19 during hospitalization at our hospital.

## Methods

### Study design and patients

The research protocol was reviewed and approved by the Ethics Committee of The First People’s Hospital of Tianmen, Hubei, China. Diagnosis and confirmation of COVID-19 were based on the guidelines published by the National Health Commission of China. Four hundred fifteen patients with confirmed COVID-19 who were admitted and hospitalized in The First People’s Hospital of Tianmen from January 14 to March 25, 2020. The age, mean (IQR) was 46.33 (34.00–57.00). Of them, 25 (6.0%) were under 18 years old, and all infected child patients have been cured, the specific data were shown in the Supplementary Table 1. All 14 cases in this study were admitted from January 18 to February 11, 2020.

### Data collection

Chronic medical histories and symptoms from onset to admission were reviewed. Clinical records, laboratory findings and radiological examinations during hospitalization were collected for analysis. Except for the results of laboratory tests (one person had incomplete laboratory test data), the rest of the results were for all 14 deaths.

### Statistical analysis

Data are presented as the mean (standard deviation, SD) or median (interquartile range, IQR) for continuous variables and number (%) for categorical variables. A Kaplan-Meier plot generated in GraphPad Prism 6 was used to show the survival curve.

## Results

### Baseline characteristics of patients who died of COVID-19

As shown in Table 1, 14 patients who died of COVID-19 in the First People’s Hospital of Tianmen were included. The mean age was 56.7 years (SD 15.3), and 8 (57.1%) patients were older than 50 years. There were more male patients (8 of 14, 57.1%). Eleven (78.6%) of these patients had a history of high-risk exposure, 2 (14.3%) of them were exposed to cases who had confirmed SARS-CoV-2 infection or were highly suspected of being infected, and 9 (64.3%) of them had a history of living in Wuhan. Regarding chronic illness, the most common diseases were chronic cardiovascular disease (7, 50.0%), hypertension (6, 42.9%), and chronic kidney disease (5, 35.7%). Notably, 4 (28.6%) patients had been undergoing regular hemodialysis treatment for years.

**Table 1:** Baseline characteristics of 14 nonsurvivors with COVID-19

Characteristics	Nonsurvivors (***n*** = 14)
Age, years, mean (SD)	56.7 (15.3)
Age range, years	
30–39	1 (7.1%)
40–49	5 (35.7%)
50–59	2 (14.3%)
60–69	2 (14.3%)
70–79	3 (21.4%)
≥ 80	1 (7.1%)
Sex	
Female	6 (42.9%)
Male	8 (57.1%)
**Exposure**	11 (78.6%)
Patients*	2 (14.3%)
Travel to Wuhan	9 (64.3%)
**Chronic illness**	11 (78.6%)
Chronic respiratory disease	0
Chronic cardiovascular disease	7 (50.0%)
Chronic kidney disease	5 (35.7%)
Chronic digestive disease	1 (7.1%)
Hypertension	6 (42.9%)
Diabetes	2 (14.3%)
Cataract	1 (7.1%)
Thoracic collapse	1 (7.1%)

Data are shown as n (%) or mean (SD), ^*^Patients with confirmed SARS-CoV-2 infection or highly suspected of being infected

### The survival curve of patients who died of COVID-19

**Fig. 1:**
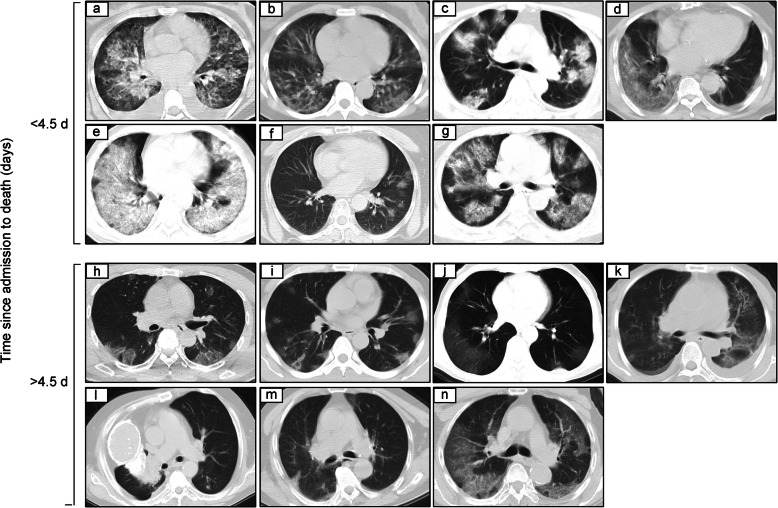
The survival curve of patients who died of COVID-19. All patients died within 42 days from the onset of illness, and the median was 11 days, IQR 6.5 to 19.5 (**a**). All patients died within 34 days of admission, and the median was 4.5 days, IQR 1.0 to 11.8. *n* = 14; IQR, interquartile range (**b**)

All 14 patients died within 42 days of the onset of illness and within 34 days after admission. The median duration from onset of symptoms to death was 11 (IQR 6.5–19.5) days (Fig. 1a). The median duration from admission to death was 4.5 (IQR 1.0–11.8) days (Fig. 1b).

### Radiological results of patients at admission

**Fig. 2:**
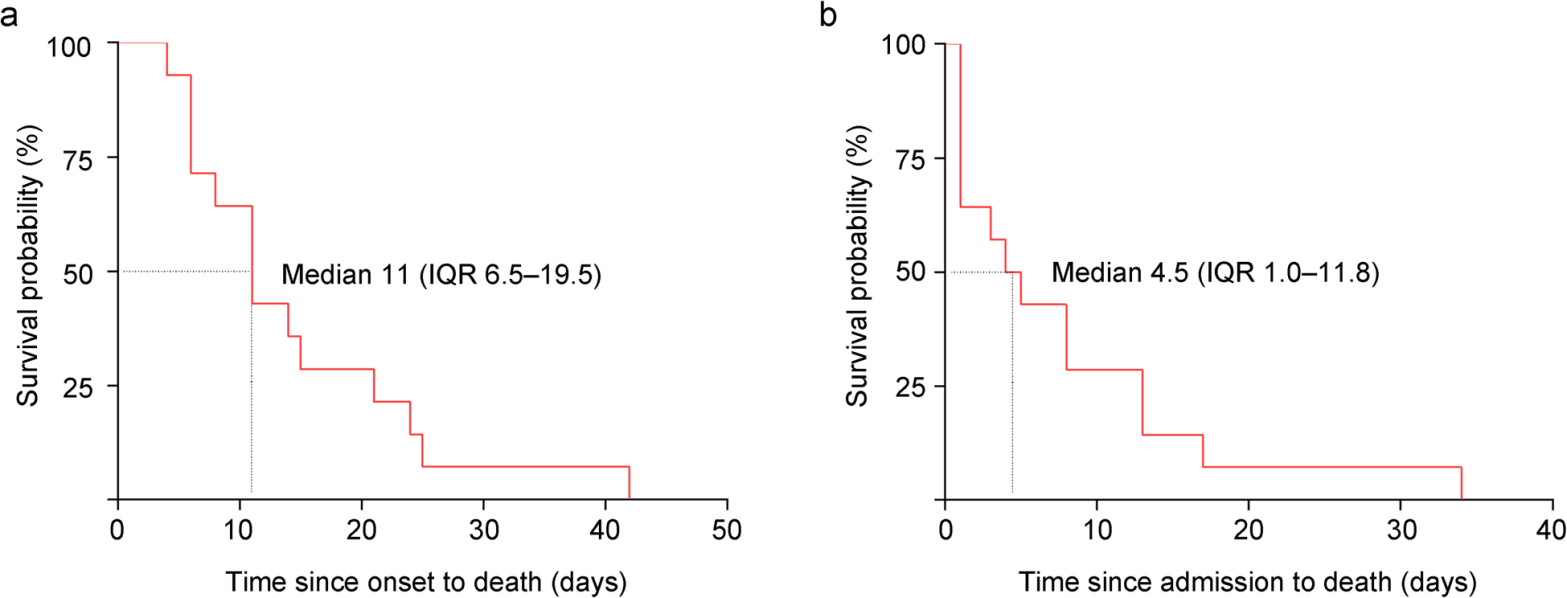
Chest CT images at admission. Transverse chest CT images from the 14 patients who died of COVID-19. Cases were divided into 2 groups according to the median duration: death within 4.5 days after admission (**a-g**) and death ≥4.5 days after admission (**h-n**)

All patients were diagnosed with pneumonia by chest computed tomography (CT) scans and were divided into two groups according to the median duration from admission to death (Fig. 2). According to the results, patients who died within 4.5 days seemed to have more severe pulmonary damage, indicated by multiple patchy ground-glass shadows, consolidation and coalescing infiltrates that pervaded the lungs (Fig. 2a-g). One patient had thoracic collapse and a reduction in the volume of the right lung, which was considered to be caused by previous tuberculosis (Fig. 2l).

### Clinical features and treatment records of the 14 nonsurvivors

As shown in Table 2, the most common symptoms at admission were cough (12, 85.7%), fever (11, 78.6%), and dyspnea (10, 71.4%). Other symptoms included sputum production (4, 28.6%), diarrhea (4, 28.6%), chest tightness (2, 14.3%), and headache (1, 7.1%). The mean systolic and diastolic blood pressure was 133.5 (16.0)/78.1 (10.7) mm Hg. For laboratory results, significantly reduced lymphocytes and elevated C-reactive protein (CRP) were also consistent with previous studies. Most patients had organ dysfunction, including 13 (92.9%) with acute respiratory distress syndrome (ARDS), 4 (28.6%) with cardiac injury, 3 (21.4%) with acute kidney injury, and 3 (21.4%) with liver dysfunction. Multiple organ dysfunction syndrome (MODS) was noted in 2 (14.3%) patients, and 2 (14.3%) patients had bacteremia. In addition, 1 (7.1%) patient had hyperglycemia, and 1 (7.1%) had fungal septicemia. All patients received a high-flow nasal cannula and antiviral treatment, including oseltamivir and lopinavir/ritonavir (Kaletra). Most patients (13, 92.9%) received antibacterial combination therapy. All patients have been intubated and ventilated, including continuous positive airway pressure (CPAP), invasive and non-invasive ventilation, most patients (13, 92.9%) were treated by non-invasive ventilator, one patient received invasive mechanical ventilation supportive therapy (tracheal intubation, tracheotomy). Ten (71.4%) patients were also treated with glucocorticoids. Five (35.7%) patients were treated with Human Immunoglobulin (pH 4) for intravenous injection. Four (28.6%) patients received aerosol inhalation treatment with α-IFN. Four (28.6%) patients received renal replacement therapy. One (7.1%) patient was treated with antifungal agents.

**Table 2:** Clinical characteristics, treatment and comorbidities of 14 nonsurvivors with COVID-19

Symptoms	Nonsurvivors (***n*** = 14)
Fever	11 (78.6%)
Cough	12 (85.7%)
Sputum production	4 (28.6%)
Dyspnea	10 (71.4%)
Myalgia	0
Chest tightness	2 (14.3%)
Headache	1 (7.1%)
Diarrhea	4 (28.6%)
**Blood pressure**	
Systolic, mm Hg	133.5 (16.0)
Diastolic, mm Hg	78.1 (10.7)
**Laboratory results**^**a**^	
Leukocytes (3.5–9.5), × 10^9^/L,	9.9 (3.0)
Lymphocyte count (1.1–3.2), × 10^9^/L,	0.6 (0.3)
Neutrophils (1.8–6.3), × 10^9^/L	9.2 (2.9)
C-reactive protein (≤6.0), × 10^9^/L	156.8 (75.5)
**Treatment**	
High-flow nasal cannula	14 (100%)
Mechanical ventilation	
Noninvasive	9 (64.3%)
Invasive	3 (21.4%)
Antiviral agents	14 (100%)
Antibacterial agents	13 (92.9%)
Antifungal agents	1 (7.1%)
Glucocorticoids	10 (71.4%)
Immunoglobulin	5 (35.7%)
α-IFN	4 (28.6%)
Renal replacement therapy	4 (28.6%)
**Comorbidities**	
Acute respiratory distress syndrome	13 (92.9%)
Acute kidney injury	3 (21.4%)
Liver dysfunction	3 (21.4%)
Cardiac injury	4 (28.6%)
Hyperglycemia	1 (7.1%)
Multiple organ dysfunction syndrome	2 (14.3%)
Bacteremia	2 (14.3%)
Fungal septicemia	1 (7.1%)

Data are shown as n (%) or mean (SD)

^a^One patient had incomplete data, *n* = 13

## Discussion

We report clinical data from 14 hospitalized deaths in patients with confirmed COVID-19 at the First People’s Hospital of Tianmen. As of March 24, 81,747 patients with COVID-19 were confirmed in China [2], of whom 496 were in Tianmen City. The COVID-19 situation in capital cities has been well reported. Without a well-equipped economic foundation, some areas near Wuhan were also facing severe damage due to the SARS-CoV-2 pandemic. However, little is known about the pandemic in small and medium-sized cities in Hubei. As the only intensive treatment center for COVID-19 patients with severe and critical illness in Tianmen, all 14 deaths in the region occurred in our hospital. Most (11, 78.6%) of them had a history of high-risk exposure, but none had been to the Huanan seafood market. Considering that Tianmen is only 150 km away from Wuhan and that a large number of return visitors arrived before Chinese New Year, the initial infections in this city were likely caused by transmission from the visiting population.

Similar to SARS-CoV and Middle Eastern respiratory syndrome (MERS)-CoV, SARS-CoV-2 can cause death by triggering acute respiratory failure [6]. In our study, more severe lung lesions were shown in the CTs of patients who died within a few days of admission, reconfirming that radiological results are conducive to evaluating the severity and outcomes of COVID-19 [7]. The initial lack of COVID-19 awareness for self-diagnosis and relatively inadequate treatment capacity may have contributed to the serious illness of the patients admitted in the early stage of the outbreak [5, 8]. A higher risk of death exists in middle-aged and older male patients. Consistent with previous research, chronic diseases and poor physical condition are also vital causes of critical illness and death in patients with COVID-19 [3, 9]. In addition, 4 (28.6%) of the patients who died had been undergoing regular hemodialysis treatment for years, suggesting higher susceptibility and worse prognosis for hemodialysis patients. Hemodialysis centers should be considered a high-risk area for COVID-19 transmission [10]. However, these patients generally have nonspecific symptoms, such as cough (12, 85.7%) and fever (11, 78.6%), on admission, which makes initial triage difficult. Regarding the laboratory tests, significant lymphocytopenia occurred in these patients. Lymphopenia caused by SARS-CoV invasion and excessive immune disorders has been proven to be a prominent feature of patient prognosis [11]. Therefore, early warning of lymphocyte changes in patients with COVID-19 and efforts to improve immune dysfunction should be performed to reduce the incidence of severe or critical illness. Thus, intravenous immunoglobulin therapy is recommended to enhance anti-infection ability. In terms of treatment, we followed national standards and administered antiviral and antibacterial agents to most patients for supportive treatment. However, no specific reagents for the clinical treatment of SARS-CoV-2 have been found to date.

Unfortunately, due to the relatively underdeveloped economy of the city where our hospital is located, we do not have the conditions or equipment to provide advanced life support, such as extracorporeal membrane oxygenation (ECMO), for critically ill patients. At the same time, hospitals in our provincial capital, Wuhan, and the surrounding cities were also nearly saturated, and were not able to transport all critical patients to superior units for better healthcare treatments in time. Therefore, the CFR of COVID-19 in our hospital and this city was the highest in the province in the early stage of the pandemic [5]. Fortunately, with the continuous support of medical staff, equipment and supplies in other regions of China, more patients have been well treated. At the same time, strict quarantine effectively reduced the severe illness rate and the CFR [5, 12] and significantly improved medical shortages caused by the outbreak. Considering that the CFR of COVID-19 is likely overestimated [13], regions with inadequate mitigation measures or hospital resources should prepare adequately to minimize the spread of infection.

## Conclusion

Given that COVID-19 is spreading worldwide, elderly infected patients with comorbidities, especially those with ARDS and poor chest CT findings, are at increased risk of death. All COVID-19 patients with severe and critical illness deserve special attention and quality medical treatment as early as possible.

## Supplementary Information

**Additional file 1: Supplementary Table 1**. Baseline characteristics of child patients with COVID-19.

## Data Availability

Data on patients with COVID-19 in this study have not been reported in any other submission by anyone else. All data generated or analyzed during this study are included in this published article, and the raw datasets, except for patient privacy used and/or analyzed during the current study are available from the corresponding author on reasonable request.
