# Correction to: Clinical characteristics of 14 COVID-19 deaths in Tianmen, China: a single-center retrospective study

**DOI:** 10.1186/s12879-021-05835-z

**Published:** 2021-01-27

**Authors:** Jijia Hu, Yingang Zhang, Wei Wang, Zhihe Tao, Juan Tian, Ning Shao, Nian Liu, Hui Wei, Hao Huang

**Affiliations:** 1 Division of Nephrology, Renmin Hospital of Wuhan University, Hubei, China; 2 Division of Medical Services, The First People’s Hospital of Tianmen, Tianmen, Hubei, China; 3 Division of Health Examination Center, The First People’s Hospital of Tianmen, Tianmen, Hubei China; 4 Director’s Office, The First People’s Hospital of Tianmen, Tianmen, Hubei China; 5 Division of Rheumatology, The First People’s Hospital of Tianmen, Tianmen, Hubei China; 6 Division of Nephrology, The First People’s Hospital of Tianmen, Tianmen, Hubei China; 7 Division of Cardiology, The First People’s Hospital of Tianmen, Tianmen, Hubei China

**Correction to: BMC Infect Dis 21, 88 (2021)**

**https://doi.org/10.1186/s12879-021-05770-z**

After publication of the original article [1], the authors identified an error in Dr. Jijia Hu’s affiliation.

The incorrect author’s affiliation is:

Division of Nephrology, Renmin Hospital of Wuhan University, Tianmen, Hubei, China.

The correct author’s affiliation is:

Division of Nephrology, Renmin Hospital of Wuhan University, Hubei, China.

The original article has been corrected.
