# Supplementary material for: Clinical characteristics of 14 COVID-19 deaths in Tianmen, China: a single-center retrospective study
Source: BMC Infect Dis. 2021 Jan 20;21:88. doi: 10.1186/s12879-021-05770-z (PMC7816151; doi:10.1186/s12879-021-05770-z)
Supplement: Supplementary file 1 — Additional file 1: Supplementary Table 1. Baseline characteristics of child patients with COVID-19. [file 12879_2021_5770_MOESM1_ESM.docx]

**Supplementary Table 1: Baseline characteristics of child patients with COVID-19**

| Characteristics | Overall | Asymptomatic | Mild | Severe |
| --- | --- | --- | --- | --- |
| Age, mean (IQR), y | 9.3 (5.0-13.0) | 8.7 (4.0-13.5) | 11.1 (10.0-13.0) | 7.5 (6.3-8.8) |
| Sex | | | | |
| Female, N (%) | 12 (48.0) | 8 (67.0) | 2 (17.0) | 2 (17.0) |
| Male, N (%) | 13 (52.0) | 8 (61.5) | 5 (38.5) | 0 |
| Outcome | | | | |
| Cured, N (%) | 25 (100.0) | 16 (64.0) | 7 (28.0) | 2 (8.0) |
| Dead | 0 | 0 | 0 | 0 |
| Total Number, N (%) | 25 (100.0) | 16 (64.0) | 7 (28.0) | 2 (8.0) |

*Note*. Data were shown as mean (IQR), median (SD) or N (%), where N is the total number of patients with available data. Abbreviations: IQR, interquartile range; SD, standard deviation.
